# A catalogue of 136 microbial draft genomes from Red Sea metagenomes

**DOI:** 10.1038/sdata.2016.50

**Published:** 2016-07-05

**Authors:** Mohamed F. Haroon, Luke R. Thompson, Donovan H. Parks, Philip Hugenholtz, Ulrich Stingl

**Affiliations:** 1 Red Sea Research Center, King Abdullah University of Science and Technology (KAUST), Thuwal 23955-6900, Saudi Arabia; 2 Department of Pediatrics, University of California, San Diego, California 92037, USA; 3 Australian Centre for Ecogenomics, School of Chemistry and Molecular Biosciences, The University of Queensland, St Lucia, Queensland 4072, Australia; 4 Institute for Molecular Bioscience, The University of Queensland, Brisbane, Queensland 4072, Australia

**Keywords:** Water microbiology, Marine biology, Genome informatics

## Abstract

Earth is expected to continue warming and the Red Sea is a model environment for understanding the effects of global warming on ocean microbiomes due to its unusually high temperature, salinity and solar irradiance. However, most microbial diversity analyses of the Red Sea have been limited to cultured representatives and single marker gene analyses, hence neglecting the substantial uncultured majority. Here, we report 136 microbial genomes (completion minus contamination is ≥50%) assembled from 45 metagenomes from eight stations spanning the Red Sea and taken from multiple depths between 10 to 500 m. Phylogenomic analysis showed that most of the retrieved genomes belong to seven different phyla of known marine microbes, but more than half representing currently uncultured species. The open-access data presented here is the largest number of Red Sea representative microbial genomes reported in a single study and will help facilitate future studies in understanding the physiology of these microorganisms and how they have adapted to the relatively harsh conditions of the Red Sea.

## Background & Summary

**Figure 1:**
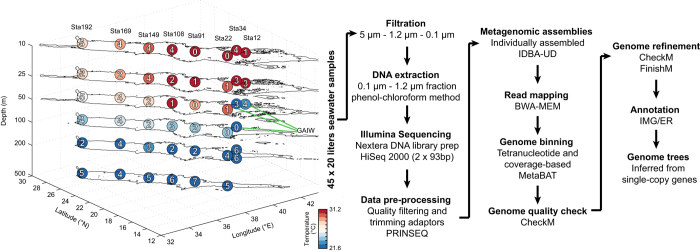
Experimental workflow for this study. The circles superimposed on the Red Sea 3D map shows the sampling points during the King Abdullah University of Science and Technology Red Sea Expedition 2011. The green lines represent the three Gulf of Aden Intermediate Water (GAIW) sampling points. The numbers within the circles represent the number of genomes recovered from each of the sample. Colors represent the high (dark red) to low (dark blue) water temperature. A total of 45 samples of 20 l each were collected and filtered through a series of filters. For this study, DNA extraction was performed on the small microbial fractions (between 0.1 to 1.2 μm). Extracted DNA was sequenced on the Illumina HiSeq 2,000 generating paired-end reads (2×93 bp). Reads from each metagenome were cleaned and assembled individually. Genomes were binned based on tetranucleotide and coverage-based method, refined and quality checked. All 136 genomes were annotated by IMG/ER and taxonomically assigned based on genome trees inferred from single-copy genes.

The Red Sea is an ideal marine environment to study microbial adaptation to physical conditions atypical of global oceans: high temperature, high salinity, and high irradiance. In late summer 2011, we undertook the King Abdullah University of Science and Technology (KAUST) Red Sea Expedition (KRSE2011) in the eastern Red Sea in order to map its diversity along environmental gradients that occur with changes in latitude, longitude, and depth^1^. This time of year is not only when temperatures and evaporation (and hence salinity) are highest, but also when a foreign water mass called the Gulf of Aden Intermediate Water (GAIW) intrudes into the Red Sea^1,2^ (Fig. 1). The GAIW brings nutrient-rich water to the Red Sea, providing nitrogen, phosphorus, and other elements to this otherwise oligotrophic sea, and is likely to introduce important microbial diversity.

Insights into the taxonomic, evolutionary, and functional diversity of the Red Sea have largely been based on studies of pure cultures^3–5^ and single marker genes such as the 16S rRNA^6,7^, or internal transcribed spacer^8^. Recently, investigations of microbial ecology have steered towards whole genome-based culture-independent methods notably single-cell genomics and metagenomics^9,10^. Single-cell genomics is an exciting field that recovers complete and partial single cell genomes from complex environments, albeit the need of specialised equipment, high cost and relatively low throughput^11–13^. Metagenomics is paving the way forward by harnessing the recent wave of sequencing technology and bioinformatics advancements to recover genomes of individual populations or populations of closely related organisms^14–16^. Application of these methods has resulted in the recovery of numerous genomes of uncultivated microorganisms that have provided surprising insights into the diversity and function of microbial communities^10,14,17–19^.

**Figure 2:**
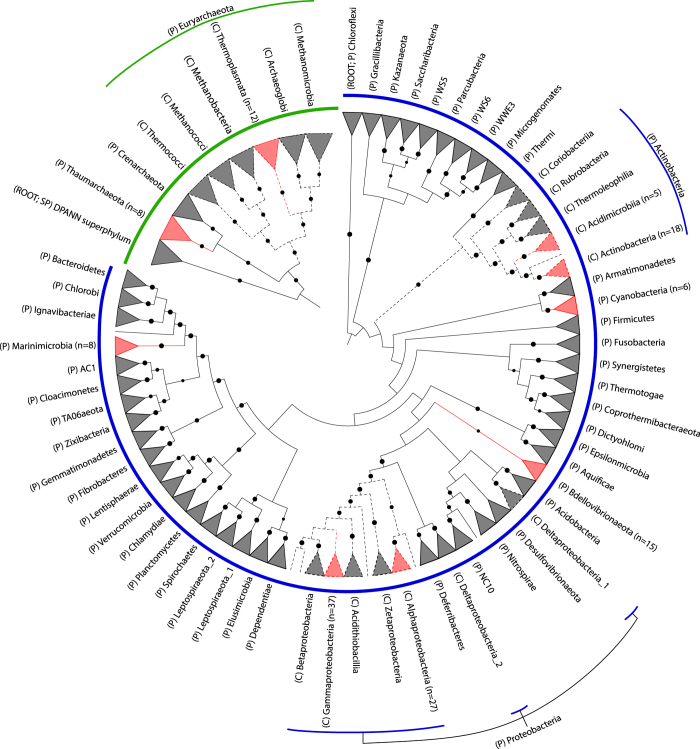
Phylogenetic trees for the archaeal (green lines; top left) and bacterial (blue lines; bottom right) domains based on 122 and 120 single-copy marker genes, respectively. The clades represented by the triangles are collapsed at the phylum (P) level except for phyla containing genomes from this study which are expanded at the class (C) level and highlighted in red. Certain phyla have genome representatives only at the phylum level (Thaumarchaeota, Marinimicrobia, Cyanobacteria, and Bdellovibrionaeota). Numbers in parentheses indicate the count of recovered genomes from a particular taxonomic level. Dashed lines indicate nodes for class level. Robustness of the tree is indicated by black circles (size of circles scaled from 80 to 100% bootstrap support values). Trees were inferred independently. The archaeal tree was rooted with the DPANN superphylum^9^ while the bacterial tree was ‘arbitrarily’ rooted with the phylum Chloroflexi^42^ but should be treated as unrooted.

During the KRSE2011, eight stations were sampled along a cruise track from south to north, capturing gradients in temperature, salinity, oxygen, and nutrients, including the unique GAIW water mass (Fig. 1 and Table 1 (available online only)). At each station, samples were collected from the surface to mesopelagic depths (10, 25, 50, 100, 200, and 500 m), except for stations 12 and 34, which had depths shallower than 500 m (Fig. 1 and Table 1 (available online only)), in order to capture a greater variation in environmental parameters and microbial diversity. Here, we successfully reconstructed 136 genomes from 45 individually assembled metagenomes (Figs 1 and 2, Tables 1 and 2 (available online only), Data Citation 1) by differential read coverage and tetranucleotide frequency methods. Of these, 43 were ‘near-complete’ with an estimated completion minus contamination of ≥90%, while the other 93 draft genomes had completion minus contamination of ≥50% (Table 2 (available online only)). To our knowledge, this is the largest number of microbial genomes from the Red Sea to be reported in a single study.

Phylogenomic analysis based on sets of single-copy marker genes universal to either the bacterial or archaeal domain showed that the 136 genomes encompassed seven phyla across these domains: Thaumarchaeota, Euryarchaeota, Actinobacteria, Cyanobacteria, Bdellovibrionaeota, Proteobacteria, and Marinimicrobia (Fig. 2 and Table 2 (available online only)). As expected, most of the recovered genomes were affiliated with known marine microorganisms such as phototrophic *Prochlorococcus*^20,21^ and *Synechococcus*^22,23^; representative of clades first discovered in the Sargasso Sea (SAR86, SAR116, SAR324 and SAR406)^24–26^; common marine bacteria in tropical biomes such as *Alteromonas macleodii*^27^; an ammonia oxidizing thaumarchaeon from the genus *Nitrosopelagicus*^28^; euryarchaeotal Marine Group II organisms reported to be abundant in surface waters^29^; members of the *Alpha-* and *Gamma-proteobacteria* such as *Aeromicrobium*, *Erythrobacter*, *Maritimibacter*, *Idiomarina*, *Marinobacter*, *Candidatus* Thioglobus (SUP05 cluster) and several unclassified *Gammaproteobacteria*, consistent with the high relative abundance of these two groups in the recent Tara Oceans survey^30^. Additionally, actinobacterial *Acidiimicrobia* and *Nocardioides* genomes thought to be responsible for secondary metabolite production in marine ecosystems^31^ were recovered from the metagenomes. An important strength of this dataset is the recovery of multiple, closely-related genomes from different stations or depths in the Red Sea (Data Citation 2). When complemented with physicochemical data^1^, genome plasticity between these organisms to confer fitness under varying conditions can be investigated in future studies.

To allow easy access to the genomes, all 136 genomes were functionally annotated and deposited into the National Centre for Biotechnology Information (NCBI) and Integrated Microbial Genomes (IMG) databases^32^. The wealth of metagenomic and genomic data described here greatly expands the repertoire of microbial genomic information from the Red Sea which might help to better understand the effects of global warming to ocean microbiomes. These datasets will also strengthen studies to better understand the drivers of marine nutrient cycling, help approaches for bioprospecting for novel thermo- and halo-philic enzymes, and allow for a better understanding of microbial adaptation strategies against high temperature, salinity and solar irradiance.

## Methods

### Metagenomic sequencing and assembly

Seawater samples were collected from eight stations and from different depths (10, 25, 50, 100, 200, and 500 m; locations are shown in Fig. 1) during summer as part of KRSE2011 (ref. 1). Genomic DNA was extracted from the 0.1–1.2 μm size fraction using an established phenol-chloroform extraction protocol^1,33^. Paired-end libraries (2×100 bp) were prepared using Nextera DNA Library Prep Kit (Illumina) and sequenced on a HiSeq 2000 (Illumina). Reads were quality checked and trimmed using PRINSEQ v0.20.4 (ref. 34) generating read lengths of ~93 bp and a total of ~10 million reads per sample with median insert sizes ranging from 183–366 bp^1^ (Data Citation 1). Trimmed metagenome reads were individually assembled (Table 1 (available online only)) using IDBA-UD v1.1.1 (ref. 35) using the ‘--pre-correction’ option. To obtain coverage profile of contigs from each metagenomic assembly, the trimmed reads were mapped back to contigs using BWA v0.7.12 (ref. 36) with the bwa-mem algorithm.

### Genome binning, refinement, and annotation

For each metagenome, genome bins were recovered based on tetranucleotide frequencies and read coverage using MetaBAT v0.26.1 (ref. 37) with default parameters. The completeness and contamination of the bins were assessed using CheckM v1.0.3 (ref. 38) using the lineage-specific workflow (Table 2 (available online only)). Bins were further refined using the CheckM ‘merge’ and ‘outliers’ commands which merge bins with complementary sets of marker genes to improve completeness and remove contigs from bins which appear to be outliers relative to reference GC and tetranucleotide distributions in order to reduce contamination^38^. The FinishM v0.0.7 (https://github.com/wwood/finishm) ‘roundup’ workflow which comprise of ‘wander’ and ‘gapfill’ modes was used to scaffold contigs together and fill gaps within individual bins. The ‘wander’ mode uses a de Bruijn graph (kmer length of 51 bp and coverage cutoff of 5) to determine contig ends which are connected while the ‘gapfill’ mode align the reads to regions of ambiguous nucleotides and replaces them with the appropriate nucleotides. Genome bins that passed the quality filter of completion minus contamination of ≥50% were submitted to IMG/ER^32^ for gene calling and functional annotation.

### Genome tree construction

The archaeal and bacterial genome trees (Fig. 2) were inferred from the concatenation of 122 and 120 proteins, respectively, identified as being present in ≥90% of the genomes in their respective domains and, when present, single-copy in ≥95% of genomes (Supplementary Tables 1 and 2). These marker genes were aligned using HMMER v3.1b1 (ref. 39) and the tree inference from the concatenated alignment with FastTree v2.1.7 (ref. 40) under the WAG+GAMMA models (Data Citation 2). Support values were determined using 100 non-parametric bootstrap replicates^41^. The archaeal tree was rooted with the DPANN (Diapherotrites, Parvarchaeota, Aenigmarchaeota, Nanohaloarchaeota, and Nanoarchaeota) superphylum in concordance with a recent large-scale phylogenomic study^9^ while the bacterial tree was ‘arbitrarily’ rooted with the phylum Chloroflexi^42^ but should be treated as unrooted. The trees were visualized in ARB^43^, annotated by iTOL^44^ and edited in Illustrator CC 2014 (Adobe).

### Code availability

All versions of third-party software and scripts used in this study are described and referenced accordingly in the Methods sub-sections for ease of access and reproducibility.

## Data Records

The raw Illumina sequencing paired-end reads (Table 1 (available online only)), 45 assembled metagenome sequences (Table 1 (available online only)) and 136 assembled genome sequences (Table 2 (available online only)), generated from the KAUST Red Sea Expedition 2011, are available from NCBI databases (Data Citation 1). The genome trees and associated fasta amino acid alignment files are available from Figshare (Data Citation 2).

## Technical Validation

To validate the completeness and contamination of the genomes, we accessed the number of marker genes present in all bacterial and archaeal genomes using CheckM^38^. The genomes were also manually cleaned from vector contamination by comparing against the UniVec core database (ftp://ftp.ncbi.nlm.nih.gov/pub/UniVec/).

## Usage Notes

The annotated genome assemblies can be downloaded and accessed via the Integrated Microbial Genomes (IMG) system (https://img.jgi.doe.gov/cgi-bin/m/main.cgi). The IMG genome IDs are provided in Table 2 (available online only).

## Additional Information

**How to cite this article:** Haroon, M. F. *et al.* A catalogue of 136 microbial draft genomes from Red Sea metagenomes. *Sci. Data* 3:160050 doi: 10.1038/sdata.2016.50 (2016).

## Supplementary Material



Supplementary Tables

## Figures and Tables

**Table 1:** Characteristics of the 45 Red Sea metagenomic samples

**Isolation source**	**Water mass**	**Date and Time**	**Assembly size (Mbps)**	**No. of scaffolds**	**Largest scaffold size (Mbps)**	**N50**	**Depth (m)**	**BioProject**	**BioSample**	**NCBI accession (assembled)**	**NCBI SRA accession (raw reads)**
Red Sea water column Station 12	Red Sea	18/09/2011 10:26	44.11	29555	0.120	2072	10	PRJNA289734	SAMN03860258	LUMR00000000	SRR2102994
Red Sea water column Station 12	Red Sea		75.83	46146	0.102	2508	25	PRJNA289734	SAMN03860259	LUMQ00000000	SRR2102995
Red Sea water column Station 12	GAIW		37.84	23158	0.157	2720	47	PRJNA289734	SAMN03860260	LUMP00000000	SRR2103006
Red Sea water column Station 22	Red Sea	19/09/2011 08:53	47.49	38508	0.060	1415	10	PRJNA289734	SAMN03860261	LUMO00000000	SRR2103017
Red Sea water column Station 22	Red Sea		40.79	27718	0.072	1973	25	PRJNA289734	SAMN03860262	LUMN00000000	SRR2103028
Red Sea water column Station 22	Red Sea		41.68	29846	0.090	1775	50	PRJNA289734	SAMN03860263	LUMM00000000	SRR2103034
Red Sea water column Station 22	Red Sea		41.80	33190	0.129	1457	100	PRJNA289734	SAMN03860264	LUML00000000	SRR2103035
Red Sea water column Station 22	Red Sea		47.47	33409	0.133	1876	200	PRJNA289734	SAMN03860265	LUMK00000000	SRR2103036
Red Sea water column Station 22	Red Sea		80.71	45186	0.179	3201	500	PRJNA289734	SAMN03860266	LUMJ00000000	SRR2103037
Red Sea water column Station 34	Red Sea	20/09/2011 04:10	77.82	46638	0.221	2646	10	PRJNA289734	SAMN03860267	LUMI00000000	SRR2103038
Red Sea water column Station 34	Red Sea		42.51	28685	0.221	2018	25	PRJNA289734	SAMN03860268	LUMH00000000	SRR2102996
Red Sea water column Station 34	GAIW		38.78	27506	0.253	1843	50	PRJNA289734	SAMN03860269	LUMG00000000	SRR2102997
Red Sea water column Station 34	GAIW		39.04	35615	0.037	1167	100	PRJNA289734	SAMN03860270	LUMF00000000	SRR2102998
Red Sea water column Station 34	Red Sea		63.41	42929	0.125	1962	200	PRJNA289734	SAMN03860271	LUME00000000	SRR2102999
Red Sea water column Station 34	Red Sea		64.65	37057	0.263	2832	258	PRJNA289734	SAMN03860272	LUMD00000000	SRR2103000
Red Sea water column Station 91	Red Sea	24/09/2011 20:54	35.42	26110	0.116	1716	10	PRJNA289734	SAMN03860273	LUMC00000000	SRR2103001
Red Sea water column Station 91	Red Sea		28.80	21238	0.079	1783	25	PRJNA289734	SAMN03860274	LUMB00000000	SRR2103002
Red Sea water column Station 91	Red Sea		21.35	18107	0.070	1293	50	PRJNA289734	SAMN03860275	LUMA00000000	SRR2103003
Red Sea water column Station 91	Red Sea		51.35	45910	0.141	1194	100	PRJNA289734	SAMN03860276	LULZ00000000	SRR2103004
Red Sea water column Station 91	Red Sea		49.04	43484	0.129	1239	200	PRJNA289734	SAMN03860277	LULY00000000	SRR2103005
Red Sea water column Station 91	Red Sea		68.61	45543	0.194	2011	500	PRJNA289734	SAMN03860278	LULX00000000	SRR2103007
Red Sea water column Station 108	Red Sea	27/09/2011 21:03	68.31	39235	1.199	2885	10	PRJNA289734	SAMN03860279	LULW00000000	SRR2103008
Red Sea water column Station 108	Red Sea		59.60	40690	0.160	1948	25	PRJNA289734	SAMN03860280	LULV00000000	SRR2103009
Red Sea water column Station 108	Red Sea		58.23	49013	0.058	1334	50	PRJNA289734	SAMN03860281	LULU00000000	SRR2103010
Red Sea water column Station 108	Red Sea		36.51	27461	0.142	1626	100	PRJNA289734	SAMN03860282	LULT00000000	SRR2103011
Red Sea water column Station 108	Red Sea		58.51	45536	0.079	1553	200	PRJNA289734	SAMN03860283	LULS00000000	SRR2103012
Red Sea water column Station 108	Red Sea		63.24	41604	0.139	2136	500	PRJNA289734	SAMN03860284	LULR00000000	SRR2103013
Red Sea water column Station 149	Red Sea	01/10/2011 05:00	56.10	31577	1.198	2984	10	PRJNA289734	SAMN03860285	LULQ00000000	SRR2103014
Red Sea water column Station 149	Red Sea		62.95	34995	0.416	3178	25	PRJNA289734	SAMN03860286	LULP00000000	SRR2103015
Red Sea water column Station 149	Red Sea		79.82	47255	0.314	2763	50	PRJNA289734	SAMN03860287	LULO00000000	SRR2103016
Red Sea water column Station 149	Red Sea		38.33	25062	0.170	2222	100	PRJNA289734	SAMN03860288	LULN00000000	SRR2103018
Red Sea water column Station 149	Red Sea		66.80	52078	0.105	1503	200	PRJNA289734	SAMN03860289	LULM00000000	SRR2103019
Red Sea water column Station 149	Red Sea		85.38	54665	0.289	2146	500	PRJNA289734	SAMN03860290	LULL00000000	SRR2103020
Red Sea water column Station 169	Red Sea	03/10/2011 04:55	99.91	54636	1.199	3019	10	PRJNA289734	SAMN03860291	LULK00000000	SRR2103021
Red Sea water column Station 169	Red Sea		83.57	45217	0.308	3232	25	PRJNA289734	SAMN03860292	LULJ00000000	SRR2103022
Red Sea water column Station 169	Red Sea		84.94	53578	0.158	2309	50	PRJNA289734	SAMN03860293	LULI00000000	SRR2103023
Red Sea water column Station 169	Red Sea		73.95	54373	0.149	1685	100	PRJNA289734	SAMN03860294	LULH00000000	SRR2103024
Red Sea water column Station 169	Red Sea		73.59	57258	0.155	1530	200	PRJNA289734	SAMN03860295	LULG00000000	SRR2103025
Red Sea water column Station 169	Red Sea		79.59	58308	0.400	1655	500	PRJNA289734	SAMN03860296	LULF00000000	SRR2103026
Red Sea water column Station 192	Red Sea	05/10/2011 10:56	98.63	57007	1.199	2789	10	PRJNA289734	SAMN03860297	LULE00000000	SRR2103027
Red Sea water column Station 192	Red Sea		58.15	34483	0.321	2666	25	PRJNA289734	SAMN03860298	LUMS00000000	SRR2103029
Red Sea water column Station 192	Red Sea		87.61	47563	1.358	3315	50	PRJNA289734	SAMN03860299	LUMT00000000	SRR2103030
Red Sea water column Station 192	Red Sea		50.63	34015	0.680	2014	100	PRJNA289734	SAMN03860300	LUMU00000000	SRR2103031
Red Sea water column Station 192	Red Sea		45.78	30314	0.295	2337	200	PRJNA289734	SAMN03860301	LUMV00000000	SRR2103032
Red Sea water column Station 192	Red Sea		73.78	44069	0.278	2528	500	PRJNA289734	SAMN03860302	LUMW00000000	SRR2103033

**Table 2:** Characteristics of the 136 genomes reported in this study

**Genome bins**	**Genome size (Mbps)**	**No. of scaffolds**	**IMG Gene count**	**GC (%)**	**Marker lineage for CheckM**	**Completeness (%)**	**Contamination (%)**	**Comp-Cont %**	**Isolation source**	**Depth (m)**	**Latitude/Longtitude**	**BioProject**	**BioSample**	**NCBI accession**	**IMG genome ID**
Acidimicrobiia bacterium REDSEA-S09_B7	2.02	226	1884	71.62	k__Bacteria (UID1453)	84.25	2.23	82.02	Red Sea water column Station 22	500	17.996 N 39.799 E	PRJNA289734	SAMN04534547	LUMX00000000	2651870138
Acidimicrobiia bacterium REDSEA-S14_B4	2.10	257	1654	71.6	k__Bacteria (UID1453)	85.86	2.56	83.3	Red Sea water column Station 34	200	18.58 N 40.743 E	PRJNA289734	SAMN04534548	LUMY00000000	2651870139
Acidimicrobiia bacterium REDSEA-S20_B6	1.76	338	2064	71.41	k__Bacteria (UID1453)	81.36	1.59	79.77	Red Sea water column Station 91	200	20.525 N 38.781 E	PRJNA289734	SAMN04534549	LUMZ00000000	2651870140
Acidimicrobiia bacterium REDSEA-S21_B10	1.73	328	2037	71.37	k__Bacteria (UID1453)	64.31	0.43	63.88	Red Sea water column Station 91	500	20.525 N 38.781 E	PRJNA289734	SAMN04534550	LUNA00000000	2651870141
Acidimicrobiia bacterium REDSEA-S33_B8N9	2.15	262	2380	71.46	k__Bacteria (UID1453)	80.48	5.56	74.92	Red Sea water column Station 149	500	23.604 N 37.054 E	PRJNA289734	SAMN04534551	LUNB00000000	2651870142
Acinetobacter sp. REDSEA-S21_B14	2.58	517	3085	39.1	f__Moraxellaceae (UID4680)	71.54	4.32	67.22	Red Sea water column Station 91	500	20.525 N 38.781 E	PRJNA289734	SAMN04534552	LUNC00000000	2651870143
Actinobacteria bacterium REDSEA-S36_B12	1.37	255	1734	62.57	o__Actinomycetales (UID1663)	60.63	0	60.63	Red Sea water column Station 169	50	25.772 N 36.116 E	PRJNA289734	SAMN04534553	LUND00000000	2651870144
Aeromicrobium sp. REDSEA-S32_B7	3.52	333	3794	71.86	o__Actinomycetales (UID1697)	95.73	6.23	89.5	Red Sea water column Station 149	200	23.604 N 37.054 E	PRJNA289734	SAMN04534555	LUNF00000000	2651870146
Aeromicrobium sp. REDSEA-S35_B1	3.56	83	3609	72.12	o__Actinomycetales (UID1697)	98.06	2.8	95.26	Red Sea water column Station 169	25	25.772 N 36.116 E	PRJNA289734	SAMN04534556	LUNG00000000	2651870147
Aeromicrobium sp. REDSEA-S38_B2	3.49	104	3608	72.12	o__Actinomycetales (UID1697)	98.53	1.9	96.63	Red Sea water column Station 169	200	25.772 N 36.116 E	PRJNA289734	SAMN04534557	LUNH00000000	2651870148
Aeromicrobium sp. REDSEA-S42_B4	3.47	54	3538	72.15	o__Actinomycetales (UID1697)	98.45	0.86	97.59	Red Sea water column Station 192	50	27.897 N 34.507 E	PRJNA289734	SAMN04534558	LUNI00000000	2651870149
Aeromicrobium sp. REDSEA-S44_B1	3.47	44	3560	72.09	o__Actinomycetales (UID1697)	98.91	0.95	97.96	Red Sea water column Station 192	200	27.897 N 34.507 E	PRJNA289734	SAMN04534559	LUNJ00000000	2651870150
Alteromonas macleodii str. REDSEA-S09_B2	4.34	102	3190	44.52	c__Gammaproteobacteria (UID4761)	98.83	0.51	98.32	Red Sea water column Station 22	500	17.996 N 39.799 E	PRJNA289734	SAMN04534560	LUNK00000000	2651870151
Alteromonas macleodii str. REDSEA-S10_B9	2.55	490	1239	44.64	c__Gammaproteobacteria (UID4761)	59.71	0.57	59.14	Red Sea water column Station 34	10	18.58 N 40.743 E	PRJNA289734	SAMN04534561	LUNL00000000	2651870152
Alteromonas macleodii str. REDSEA-S12_B5	2.64	514	2355	43.98	c__Gammaproteobacteria (UID4761)	59.42	2.39	57.03	Red Sea water column Station 34	50	18.58 N 40.743 E	PRJNA289734	SAMN04534562	LUNM00000000	2651870153
Alteromonas macleodii str. REDSEA-S14_B11	3.11	543	2135	44.58	c__Gammaproteobacteria (UID4761)	73.75	1.24	72.51	Red Sea water column Station 34	200	18.58 N 40.743 E	PRJNA289734	SAMN04534563	LUNN00000000	2651870154
Alteromonas macleodii str. REDSEA-S15_B11	3.79	457	3227	44.59	c__Gammaproteobacteria (UID4761)	89.9	1.25	88.65	Red Sea water column Station 34	258	18.58 N 40.743 E	PRJNA289734	SAMN04534564	LUNO00000000	2651870155
Candidatus Marinimicrobia (SAR406 cluster) bacterium REDSEA-S14_B6	1.43	234	2855	54.35	k__Bacteria (UID2495)	72.35	0.18	72.17	Red Sea water column Station 34	200	18.58 N 40.743 E	PRJNA289734	SAMN04534578	LUOC00000000	2651870223
Candidatus Marinimicrobia (SAR406 cluster) bacterium REDSEA-S15_B10	1.65	233	4044	54.08	k__Bacteria (UID2495)	71.03	0.1	70.93	Red Sea water column Station 34	258	18.58 N 40.743 E	PRJNA289734	SAMN04534579	LUOD00000000	2651870224
Candidatus Marinimicrobia (SAR406 cluster) bacterium REDSEA-S15_B13	1.48	201	3478	41.84	k__Bacteria (UID2495)	71.59	3.3	68.29	Red Sea water column Station 34	258	18.58 N 40.743 E	PRJNA289734	SAMN04534580	LUOE00000000	2651870225
Candidatus Marinimicrobia (SAR406 cluster) bacterium REDSEA-S27_B1N12	1.36	280	1643	51.44	k__Bacteria (UID2495)	60.19	2.2	57.99	Red Sea water column Station 108	500	22.046 N 37.929 E	PRJNA289734	SAMN04534581	LUOF00000000	2651870226
Candidatus Marinimicrobia (SAR406 cluster) bacterium REDSEA-S33_B13	1.27	264	1462	54.33	k__Bacteria (UID2495)	56.03	1.65	54.38	Red Sea water column Station 149	500	23.604 N 37.054 E	PRJNA289734	SAMN04534582	LUOG00000000	2651870227
Candidatus Marinimicrobia (SAR406 cluster) bacterium REDSEA-S38_B13	1.15	217	1313	41.56	k__Bacteria (UID2495)	58.34	1.2	57.14	Red Sea water column Station 169	200	25.772 N 36.116 E	PRJNA289734	SAMN04534583	LUOH00000000	2651870228
Candidatus Marinimicrobia (SAR406 cluster) bacterium REDSEA-S39_B11	1.09	225	1203	54.56	k__Bacteria (UID2495)	52.97	0.1	52.87	Red Sea water column Station 169	500	25.772 N 36.116 E	PRJNA289734	SAMN04534584	LUOI00000000	2651870229
Candidatus Marinimicrobia (SAR406 cluster) bacterium REDSEA-S39_B7	0.24	15	229	38.13	k__Bacteria (UID2495)	78.5	6.89	71.61	Red Sea water column Station 169	500	25.772 N 36.116 E	PRJNA289734	SAMN04534585	LUOJ00000000	2651870230
Candidatus Thioglobus (SUP05 cluster) sp. REDSEA-S03_B1	1.58	97	2264	38.39	p__Proteobacteria (UID3880)	87.92	3.86	84.06	Red Sea water column Station 12	47	17.662 N 40.905 E	PRJNA289734	SAMN04534667	LURM00000000	2651870213
Candidatus Thioglobus (SUP05 cluster) sp. REDSEA-S12_B1	1.61	77	1540	38.35	p__Proteobacteria (UID3880)	88.91	1.32	87.59	Red Sea water column Station 34	50	18.58 N 40.743 E	PRJNA289734	SAMN04534668	LURN00000000	2651870214
Candidatus Thioglobus (SUP05 cluster) sp. REDSEA-S14_B12	1.83	262	2336	39.85	p__Proteobacteria (UID3880)	67.29	7.86	59.43	Red Sea water column Station 34	200	18.58 N 40.743 E	PRJNA289734	SAMN04534669	LURO00000000	2651870215
Erythrobacter sp. REDSEA-S22_B4	2.69	10	2897	63.73	o__Sphingomonadales (UID3310)	99.93	0.43	99.5	Red Sea water column Station 108	10	22.046 N 37.929 E	PRJNA289734	SAMN04534565	LUNP00000000	2651870156
Erythrobacter sp. REDSEA-S28_B2	2.69	8	2894	63.71	o__Sphingomonadales (UID3310)	99.9	0.43	99.47	Red Sea water column Station 149	10	23.604 N 37.054 E	PRJNA289734	SAMN04534566	LUNQ00000000	2654587888
Erythrobacter sp. REDSEA-S34_B3	2.69	7	2893	63.73	o__Sphingomonadales (UID3310)	99.93	0.43	99.5	Red Sea water column Station 169	10	25.772 N 36.116 E	PRJNA289734	SAMN04534567	LUNR00000000	2654587889
Erythrobacter sp. REDSEA-S36_B6	2.93	99	3035	63.57	o__Sphingomonadales (UID3310)	97.09	2.53	94.56	Red Sea water column Station 169	50	25.772 N 36.116 E	PRJNA289734	SAMN04534568	LUNS00000000	2654587890
Erythrobacter sp. REDSEA-S37_B3	2.79	94	2968	63.54	o__Sphingomonadales (UID3310)	97.59	1	96.59	Red Sea water column Station 169	100	25.772 N 36.116 E	PRJNA289734	SAMN04534569	LUNT00000000	2654587891
Erythrobacter sp. REDSEA-S40_B1	2.69	7	2897	63.72	o__Sphingomonadales (UID3310)	99.93	0.43	99.5	Red Sea water column Station 192	10	27.897 N 34.507 E	PRJNA289734	SAMN04534570	LUNU00000000	2654587892
Erythrobacter sp. REDSEA-S41_B1	2.93	23	2933	63.59	o__Sphingomonadales (UID3310)	99.54	0.63	98.91	Red Sea water column Station 192	25	27.897 N 34.507 E	PRJNA289734	SAMN04534571	LUNV00000000	2654587893
Erythrobacter sp. REDSEA-S42_B5	2.68	12	2905	63.72	o__Sphingomonadales (UID3310)	99.83	0.43	99.4	Red Sea water column Station 192	50	27.897 N 34.507 E	PRJNA289734	SAMN04534572	LUNW00000000	2654587894
Erythrobacter sp. REDSEA-S43_B2	2.87	18	2918	63.64	o__Sphingomonadales (UID3310)	99.84	0.94	98.9	Red Sea water column Station 192	100	27.897 N 34.507 E	PRJNA289734	SAMN04534573	LUNX00000000	2651870157
Erythrobacter sp. REDSEA-S45_B7	2.89	285	3139	63.54	o__Sphingomonadales (UID3310)	89.24	1.25	87.99	Red Sea water column Station 192	500	27.897 N 34.507 E	PRJNA289734	SAMN04534574	LUNY00000000	2651870158
Idiomarina sp. REDSEA-S21_B4	2.26	60	2451	47.2	c__Gammaproteobacteria (UID4761)	95.29	0.42	94.87	Red Sea water column Station 91	500	20.525 N 38.781 E	PRJNA289734	SAMN04534575	LUNZ00000000	2651870159
Idiomarina sp. REDSEA-S27_B4	2.38	57	2550	47.27	c__Gammaproteobacteria (UID4761)	98.14	0.74	97.4	Red Sea water column Station 108	500	22.046 N 37.929 E	PRJNA289734	SAMN04534576	LUOA00000000	2651870160
Marine group II euryarchaeote REDSEA-S03_B6	1.32	210	1400	45.47	p__Euryarchaeota (UID3)	65.01	2.3	62.71	Red Sea water column Station 12	47	17.662 N 40.905 E	PRJNA289734	SAMN04534670	LURP00000000	2651870292
Marine group II euryarchaeote REDSEA-S10_B2	1.23	29	3698	50.45	p__Euryarchaeota (UID3)	76.13	1.2	74.93	Red Sea water column Station 34	10	18.58 N 40.743 E	PRJNA289734	SAMN04534671	LURQ00000000	2651870293
Marine group II euryarchaeote REDSEA-S11_B3N4	1.30	35	2197	50.98	p__Euryarchaeota (UID3)	81.96	0	81.96	Red Sea water column Station 34	25	18.58 N 40.743 E	PRJNA289734	SAMN04534672	LURR00000000	2651870294
Marine group II euryarchaeote REDSEA-S19_B7N8	1.17	155	1796	52.04	p__Euryarchaeota (UID3)	70.4	0.8	69.6	Red Sea water column Station 91	100	20.525 N 38.781 E	PRJNA289734	SAMN04534673	LURS00000000	2651870295
Marine group II euryarchaeote REDSEA-S25_B4N5	1.10	167	1272	52.14	p__Euryarchaeota (UID3)	58.43	0.27	58.16	Red Sea water column Station 108	100	22.046 N 37.929 E	PRJNA289734	SAMN04534674	LURT00000000	2651870296
Marine group II euryarchaeote REDSEA-S29_B8N9	1.10	131	1244	50.19	p__Euryarchaeota (UID3)	59.47	0	59.47	Red Sea water column Station 149	25	23.604 N 37.054 E	PRJNA289734	SAMN04534675	LURU00000000	2651870297
Marine group II euryarchaeote REDSEA-S30_B12	1.27	137	1374	36.9	p__Euryarchaeota (UID3)	67.79	0.8	66.99	Red Sea water column Station 149	50	23.604 N 37.054 E	PRJNA289734	SAMN04534676	LURV00000000	2651870298
Marine group II euryarchaeote REDSEA-S37_B2N9	1.21	6	155	49.08	p__Euryarchaeota (UID3)	76.47	0.06	76.41	Red Sea water column Station 169	100	25.772 N 36.116 E	PRJNA289734	SAMN04534677	LURW00000000	2651870299
Marine group II euryarchaeote REDSEA-S40_B11N13	1.24	118	1300	50.09	p__Euryarchaeota (UID3)	71.96	0	71.96	Red Sea water column Station 192	10	27.897 N 34.507 E	PRJNA289734	SAMN04534678	LURX00000000	2651870300
Marine group II euryarchaeote REDSEA-S41_B6	1.13	122	1216	49.97	p__Euryarchaeota (UID3)	71.35	1.92	69.43	Red Sea water column Station 192	25	27.897 N 34.507 E	PRJNA289734	SAMN04534679	LURY00000000	2651870301
Marine group II euryarchaeote REDSEA-S42_B7	1.19	43	1202	49.72	p__Euryarchaeota (UID3)	75.73	0	75.73	Red Sea water column Station 192	50	27.897 N 34.507 E	PRJNA289734	SAMN04534680	LURZ00000000	2651870302
Marine group II euryarchaeote REDSEA-S43_B8	1.10	105	1213	50.28	p__Euryarchaeota (UID3)	72.13	0	72.13	Red Sea water column Station 192	100	27.897 N 34.507 E	PRJNA289734	SAMN04534681	LUSA00000000	2651870303
Marinobacter sp. REDSEA-S15_B16	2.89	527	2873	57.64	c__Gammaproteobacteria (UID4444)	72.81	0.89	71.92	Red Sea water column Station 34	258	18.58 N 40.743 E	PRJNA289734	SAMN04534586	LUOK00000000	2651870218
Marinobacter sp. REDSEA-S21_B2N3	4.37	262	4343	57.01	c__Gammaproteobacteria (UID4444)	88.75	2.59	86.16	Red Sea water column Station 91	500	20.525 N 38.781 E	PRJNA289734	SAMN04534587	LUOL00000000	2651870219
Marinobacter sp. REDSEA-S27_B10	3.27	500	3781	57.24	k__Bacteria (UID203)	70	0	70	Red Sea water column Station 108	500	22.046 N 37.929 E	PRJNA289734	SAMN04534588	LUOM00000000	2651870220
Maritimibacter sp. REDSEA-S28_B5	4.25	122	4632	64.32	f__Rhodobacteraceae (UID3356)	97.93	0.68	97.25	Red Sea water column Station 149	10	23.604 N 37.054 E	PRJNA289734	SAMN04534590	LUOO00000000	2651870216
Maritimibacter sp. REDSEA-S40_B3	3.87	28	4511	64.35	f__Rhodobacteraceae (UID3356)	99.7	0.68	99.02	Red Sea water column Station 192	10	27.897 N 34.507 E	PRJNA289734	SAMN04534591	LUOP00000000	2651870217
Moraxellaceae bacterium REDSEA-S29_B6	2.23	154	2327	41.94	c__Gammaproteobacteria (UID4201)	91.59	0	91.59	Red Sea water column Station 149	25	23.604 N 37.054 E	PRJNA289734	SAMN04534592	LUOQ00000000	2651870134
Moraxellaceae bacterium REDSEA-S32_B1	2.38	80	2272	42.07	c__Gammaproteobacteria (UID4201)	98.13	0	98.13	Red Sea water column Station 149	200	23.604 N 37.054 E	PRJNA289734	SAMN04534593	LUOR00000000	2654587887
Moraxellaceae bacterium REDSEA-S35_B9	1.60	306	1900	41.91	c__Gammaproteobacteria (UID4201)	71.1	0.77	70.33	Red Sea water column Station 169	25	25.772 N 36.116 E	PRJNA289734	SAMN04534594	LUOS00000000	2651870137
Moraxellaceae bacterium REDSEA-S38_B3	2.41	100	2293	41.98	c__Gammaproteobacteria (UID4201)	97.33	0	97.33	Red Sea water column Station 169	200	25.772 N 36.116 E	PRJNA289734	SAMN04534595	LUOT00000000	2651870132
Moraxellaceae bacterium REDSEA-S42_B15	1.88	270	2124	41.99	c__Gammaproteobacteria (UID4201)	78.54	0.57	77.97	Red Sea water column Station 192	50	27.897 N 34.507 E	PRJNA289734	SAMN04534596	LUOU00000000	2651870135
Moraxellaceae bacterium REDSEA-S44_B2	2.35	95	2324	42.04	c__Gammaproteobacteria (UID4201)	97.13	1.15	95.98	Red Sea water column Station 192	200	27.897 N 34.507 E	PRJNA289734	SAMN04534597	LUOV00000000	2651870133
Moraxellaceae bacterium REDSEA-S45_B11	1.71	287	1938	42.04	c__Gammaproteobacteria (UID4201)	78.09	2.04	76.05	Red Sea water column Station 192	500	27.897 N 34.507 E	PRJNA289734	SAMN04534598	LUOW00000000	2651870136
Nitrosopelagicus sp. REDSEA-S08_B1	0.58	92	1823	35.43	k__Archaea (UID2)	58.9	5.83	53.07	Red Sea water column Station 22	200	17.996 N 39.799 E	PRJNA289734	SAMN04534599	LUOX00000000	2651870205
Nitrosopelagicus sp. REDSEA-S19_B12N3	1.51	307	1226	36.28	k__Archaea (UID2)	85.74	5.34	80.4	Red Sea water column Station 91	100	20.525 N 38.781 E	PRJNA289734	SAMN04534600	LUOY00000000	2651870206
Nitrosopelagicus sp. REDSEA-S25_B3	0.89	116	1294	34.1	k__Archaea (UID2)	74.76	1.94	72.82	Red Sea water column Station 108	100	22.046 N 37.929 E	PRJNA289734	SAMN04534601	LUOZ00000000	2651870207
Nitrosopelagicus sp. REDSEA-S27_B13N2	1.51	273	2009	37.26	k__Archaea (UID2)	69.84	4.85	64.99	Red Sea water column Station 108	500	22.046 N 37.929 E	PRJNA289734	SAMN04534602	LUPA00000000	2651870208
Nitrosopelagicus sp. REDSEA-S31_B2	1.01	86	1387	34.05	k__Archaea (UID2)	92.64	2.02	90.62	Red Sea water column Station 149	100	23.604 N 37.054 E	PRJNA289734	SAMN04534603	LUPB00000000	2651870209
Nitrosopelagicus sp. REDSEA-S32_B2	0.45	55	604	33.9	k__Archaea (UID2)	53.4	1.94	51.46	Red Sea water column Station 149	200	23.604 N 37.054 E	PRJNA289734	SAMN04534604	LUPC00000000	2651870210
Nitrosopelagicus sp. REDSEA-S37_B6	0.87	104	1233	34.05	k__Archaea (UID2)	89.81	1.94	87.87	Red Sea water column Station 169	100	25.772 N 36.116 E	PRJNA289734	SAMN04534605	LUPD00000000	2651870211
Nitrosopelagicus sp. REDSEA-S43_B1	0.98	115	1403	33.94	k__Archaea (UID2)	84.95	4.53	80.42	Red Sea water column Station 192	100	27.897 N 34.507 E	PRJNA289734	SAMN04534606	LUPE00000000	2651870212
Nocardioides sp. REDSEA-S22_B2	3.76	94	4050	71.84	o__Actinomycetales (UID1697)	94.43	1.38	93.05	Red Sea water column Station 108	10	22.046 N 37.929 E	PRJNA289734	SAMN04534607	LUPF00000000	2651870231
Nocardioides sp. REDSEA-S25_B9	2.25	485	2643	71.44	o__Actinomycetales (UID1697)	57.78	1.17	56.61	Red Sea water column Station 108	100	22.046 N 37.929 E	PRJNA289734	SAMN04534608	LUPG00000000	2651870232
Nocardioides sp. REDSEA-S28_B4	3.90	313	4194	71.71	o__Actinomycetales (UID1697)	94.91	3.54	91.37	Red Sea water column Station 149	10	23.604 N 37.054 E	PRJNA289734	SAMN04534609	LUPH00000000	2651870233
Nocardioides sp. REDSEA-S30_B4	3.68	104	3857	71.94	o__Actinomycetales (UID1697)	96.89	0.86	96.03	Red Sea water column Station 149	50	23.604 N 37.054 E	PRJNA289734	SAMN04534610	LUPI00000000	2651870234
Nocardioides sp. REDSEA-S31_B4	3.44	387	3841	71.68	o__Actinomycetales (UID1697)	83.54	1.21	82.33	Red Sea water column Station 149	100	23.604 N 37.054 E	PRJNA289734	SAMN04534611	LUPJ00000000	2651870235
Nocardioides sp. REDSEA-S33_B3	3.56	61	3796	72.09	o__Actinomycetales (UID1697)	98.19	0	98.19	Red Sea water column Station 149	500	23.604 N 37.054 E	PRJNA289734	SAMN04534612	LUPK00000000	2651870236
Nocardioides sp. REDSEA-S34_B5	4.17	291	4427	71.75	o__Actinomycetales (UID1697)	94.13	4.13	90	Red Sea water column Station 169	10	25.772 N 36.116 E	PRJNA289734	SAMN04534613	LUPL00000000	2651870237
Nocardioides sp. REDSEA-S36_B10	2.34	439	2744	71.08	o__Actinomycetales (UID1697)	59.33	1.26	58.07	Red Sea water column Station 169	50	25.772 N 36.116 E	PRJNA289734	SAMN04534614	LUPM00000000	2651870238
Nocardioides sp. REDSEA-S37_B12	2.85	503	3278	71.42	o__Actinomycetales (UID1697)	77.85	0.6	77.25	Red Sea water column Station 169	100	25.772 N 36.116 E	PRJNA289734	SAMN04534615	LUPN00000000	2651870239
Nocardioides sp. REDSEA-S39_B2	3.48	56	3796	72.16	o__Actinomycetales (UID1697)	97.67	0.05	97.62	Red Sea water column Station 169	500	25.772 N 36.116 E	PRJNA289734	SAMN04534616	LUPO00000000	2651870240
Nocardioides sp. REDSEA-S40_B4	3.70	116	3967	71.95	o__Actinomycetales (UID1697)	91.19	1.11	90.08	Red Sea water column Station 192	10	27.897 N 34.507 E	PRJNA289734	SAMN04534617	LUPP00000000	2651870241
Nocardioides sp. REDSEA-S43_B3	3.68	164	3948	71.9	o__Actinomycetales (UID1697)	96.07	0.35	95.72	Red Sea water column Station 192	100	27.897 N 34.507 E	PRJNA289734	SAMN04534618	LUPQ00000000	2651870242
Prochlorococcus sp. REDSEA-S17_B1	1.07	152	2041	30.99	p__Cyanobacteria (UID2143)	63.12	4.65	58.47	Red Sea water column Station 91	25	20.525 N 38.781 E	PRJNA289734	SAMN04534620	LUPS00000000	2651870162
Prochlorococcus sp. REDSEA-S22_B1	1.01	113	1338	31.11	p__Cyanobacteria (UID2143)	60.42	7.38	53.04	Red Sea water column Station 108	10	22.046 N 37.929 E	PRJNA289734	SAMN04534621	LUPT00000000	2651870163
Prochlorococcus sp. REDSEA-S23_B1	1.06	123	1381	30.87	p__Cyanobacteria (UID2143)	61.61	7.2	54.41	Red Sea water column Station 108	25	22.046 N 37.929 E	PRJNA289734	SAMN04534622	LUPU00000000	2651870164
Prochlorococcus sp. REDSEA-S28_B1	0.93	101	1181	31.35	p__Cyanobacteria (UID2143)	55.19	4.71	50.48	Red Sea water column Station 149	10	23.604 N 37.054 E	PRJNA289734	SAMN04534623	LUPV00000000	2651870165
Rhodobacteraceae bacterium REDSEA-S02_B3	2.07	113	2195	39.74	f__Rhodobacteraceae (UID3340)	80.46	1.04	79.42	Red Sea water column Station 12	25	17.662 N 40.905 E	PRJNA289734	SAMN04534625	LUPX00000000	2651870273
Rhodobacteraceae bacterium REDSEA-S03_B4	2.03	202	1380	39.69	f__Rhodobacteraceae (UID3340)	77.8	4.86	72.94	Red Sea water column Station 12	47	17.662 N 40.905 E	PRJNA289734	SAMN04534626	LUPY00000000	2651870274
Rhodobacteraceae bacterium REDSEA-S11_B6	1.89	192	2900	39.63	k__Bacteria (UID203)	77.43	8.62	68.81	Red Sea water column Station 34	25	18.58 N 40.743 E	PRJNA289734	SAMN04534628	LUQA00000000	2651870277
Rhodobacteraceae bacterium REDSEA-S29_B10	1.79	357	2262	40.1	f__Rhodobacteraceae (UID3340)	57.51	3.99	53.52	Red Sea water column Station 149	25	23.604 N 37.054 E	PRJNA289734	SAMN04534629	LUQB00000000	2651870278
Rhodobacteraceae bacterium REDSEA-S34_B6	2.41	111	2606	40.44	f__Rhodobacteraceae (UID3340)	89.51	1.57	87.94	Red Sea water column Station 169	10	25.772 N 36.116 E	PRJNA289734	SAMN04534630	LUQC00000000	2651870280
SAR116 cluster alphaproteobacterium REDSEA-S02_B12	1.51	247	2215	63.03	c__Alphaproteobacteria (UID3305)	74.63	0.44	74.19	Red Sea water column Station 12	25	17.662 N 40.905 E	PRJNA289734	SAMN04534631	LUQD00000000	2654587886
SAR116 cluster alphaproteobacterium REDSEA-S10_B10N8	1.58	247	2222	62.96	c__Alphaproteobacteria (UID3305)	78.7	0	78.7	Red Sea water column Station 34	10	18.58 N 40.743 E	PRJNA289734	SAMN04534632	LUQE00000000	2651870131
SAR324 cluster deltaproteobacterium REDSEA-S05_B4	1.75	357	2060	46.78	k__Bacteria (UID3187)	54.11	0.94	53.17	Red Sea water column Station 22	25	17.996 N 39.799 E	PRJNA289734	SAMN04534633	LUQF00000000	2654587902
SAR324 cluster deltaproteobacterium REDSEA-S06_B4	1.73	373	873	47.26	k__Bacteria (UID3187)	54.22	0.05	54.17	Red Sea water column Station 22	50	17.996 N 39.799 E	PRJNA289734	SAMN04534634	LUQG00000000	2651870251
SAR324 cluster deltaproteobacterium REDSEA-S08_B7	2.12	328	1504	43.26	k__Bacteria (UID2495)	55.09	4.09	51	Red Sea water column Station 22	200	17.996 N 39.799 E	PRJNA289734	SAMN04534635	LUQH00000000	2651870252
SAR324 cluster deltaproteobacterium REDSEA-S09_B3	3.35	83	1823	42.85	k__Bacteria (UID3187)	92.1	0	92.1	Red Sea water column Station 22	500	17.996 N 39.799 E	PRJNA289734	SAMN04534636	LUQI00000000	2651870253
SAR324 cluster deltaproteobacterium REDSEA-S10_B5	3.49	290	2962	47.12	k__Bacteria (UID3187)	90.84	0	90.84	Red Sea water column Station 34	10	18.58 N 40.743 E	PRJNA289734	SAMN04270322	LNZD00000000	2651870254
SAR324 cluster deltaproteobacterium REDSEA-S11_B7	2.54	416	1743	47.48	k__Bacteria (UID3187)	73.12	3.78	69.34	Red Sea water column Station 34	25	18.58 N 40.743 E	PRJNA289734	SAMN04534638	LUQJ00000000	2651870255
SAR324 cluster deltaproteobacterium REDSEA-S14_B10	1.95	455	3426	42.39	k__Bacteria (UID3187)	62	3.12	58.88	Red Sea water column Station 34	200	18.58 N 40.743 E	PRJNA289734	SAMN04534639	LUQK00000000	2651870256
SAR324 cluster deltaproteobacterium REDSEA-S15_B6	2.79	191	1471	42.4	k__Bacteria (UID3187)	86.18	1.73	84.45	Red Sea water column Station 34	258	18.58 N 40.743 E	PRJNA289734	SAMN04534640	LUQL00000000	2651870257
SAR324 cluster deltaproteobacterium REDSEA-S21_B5	3.05	128	3061	42.86	k__Bacteria (UID3187)	89.51	0.22	89.29	Red Sea water column Station 91	500	20.525 N 38.781 E	PRJNA289734	SAMN04534641	LUQM00000000	2651870258
SAR324 cluster deltaproteobacterium REDSEA-S26_B7	2.26	415	2573	42.53	k__Bacteria (UID3187)	69.56	1.05	68.51	Red Sea water column Station 108	200	22.046 N 37.929 E	PRJNA289734	SAMN04534642	LUQN00000000	2651870259
SAR324 cluster deltaproteobacterium REDSEA-S27_B3	3.30	80	3134	42.85	k__Bacteria (UID3187)	92.1	0	92.1	Red Sea water column Station 108	500	22.046 N 37.929 E	PRJNA289734	SAMN04534643	LUQO00000000	2651870260
SAR324 cluster deltaproteobacterium REDSEA-S33_B4	3.12	94	3069	42.82	k__Bacteria (UID3187)	92.1	0	92.1	Red Sea water column Station 149	500	23.604 N 37.054 E	PRJNA289734	SAMN04534644	LUQP00000000	2651870261
SAR324 cluster deltaproteobacterium REDSEA-S36_B13	1.37	323	1640	46.89	k__Bacteria (UID3187)	51.76	1.68	50.08	Red Sea water column Station 169	50	25.772 N 36.116 E	PRJNA289734	SAMN04534645	LUQQ00000000	2651870262
SAR324 cluster deltaproteobacterium REDSEA-S39_B5	3.04	266	3057	43	k__Bacteria (UID3187)	88.88	2.6	86.28	Red Sea water column Station 169	500	25.772 N 36.116 E	PRJNA289734	SAMN04534646	LUQR00000000	2651870263
SAR324 cluster deltaproteobacterium REDSEA-S45_B3	3.17	89	3038	42.89	k__Bacteria (UID3187)	92.1	0	92.1	Red Sea water column Station 192	500	27.897 N 34.507 E	PRJNA289734	SAMN04534647	LUQS00000000	2651870264
SAR86 cluster gammaproteobacterium REDSEA-S08_B3	1.56	177	2329	36.99	c__Gammaproteobacteria (UID4443)	61.92	4.16	57.76	Red Sea water column Station 22	200	17.996 N 39.799 E	PRJNA289734	SAMN04534648	LUQT00000000	2651870265
SAR86 cluster gammaproteobacterium REDSEA-S09_B4	1.67	107	1534	37.05	c__Gammaproteobacteria (UID4443)	68.13	2.49	65.64	Red Sea water column Station 22	500	17.996 N 39.799 E	PRJNA289734	SAMN04534649	LUQU00000000	2651870266
SAR86 cluster gammaproteobacterium REDSEA-S20_B12N4	1.70	364	2043	38.32	c__Gammaproteobacteria (UID4201)	63.97	8.96	55.01	Red Sea water column Station 91	200	20.525 N 38.781 E	PRJNA289734	SAMN04534650	LUQV00000000	2651870267
SAR86 cluster gammaproteobacterium REDSEA-S21_B7	1.52	187	1781	37.04	c__Gammaproteobacteria (UID4201)	75.21	2.87	72.34	Red Sea water column Station 91	500	20.525 N 38.781 E	PRJNA289734	SAMN04534651	LUQW00000000	2651870268
SAR86 cluster gammaproteobacterium REDSEA-S45_B6	1.57	123	1781	37	k__Bacteria (UID203)	83.07	6.58	76.49	Red Sea water column Station 192	500	27.897 N 34.507 E	PRJNA289734	SAMN04534654	LUQZ00000000	2651870271
Sphingopyxis sp. REDSEA-S22_B5	3.06	103	3606	65.03	o__Sphingomonadales (UID3310)	98.03	0.73	97.3	Red Sea water column Station 108	10	22.046 N 37.929 E	PRJNA289734	SAMN04534655	LURA00000000	2651870196
Sphingopyxis sp. REDSEA-S23_B6	3.24	118	3560	65.08	o__Sphingomonadales (UID3310)	96.02	0.34	95.68	Red Sea water column Station 108	25	22.046 N 37.929 E	PRJNA289734	SAMN04534656	LURB00000000	2651870197
Sphingopyxis sp. REDSEA-S24_B7	1.75	439	2156	65.3	o__Sphingomonadales (UID3310)	52.05	1.47	50.58	Red Sea water column Station 108	50	22.046 N 37.929 E	PRJNA289734	SAMN04534657	LURC00000000	2651870198
Sphingopyxis sp. REDSEA-S29_B3	3.46	43	3540	65.19	o__Sphingomonadales (UID3310)	98.64	0.68	97.96	Red Sea water column Station 149	25	23.604 N 37.054 E	PRJNA289734	SAMN04534659	LURE00000000	2651870200
Sphingopyxis sp. REDSEA-S34_B10	1.75	399	2178	65.03	o__Sphingomonadales (UID3310)	50.93	0.76	50.17	Red Sea water column Station 169	10	25.772 N 36.116 E	PRJNA289734	SAMN04534660	LURF00000000	2651870201
Sphingopyxis sp. REDSEA-S38_B16	2.14	337	2485	64.84	o__Sphingomonadales (UID3310)	68.27	3.72	64.55	Red Sea water column Station 169	200	25.772 N 36.116 E	PRJNA289734	SAMN04534661	LURG00000000	2651870202
Sphingopyxis sp. REDSEA-S40_B6	3.45	73	3558	65.18	o__Sphingomonadales (UID3310)	97.42	0.51	96.91	Red Sea water column Station 192	10	27.897 N 34.507 E	PRJNA289734	SAMN04534662	LURH00000000	2651870203
Sphingopyxis sp. REDSEA-S42_B3	2.91	11	3455	65.26	o__Sphingomonadales (UID3310)	99.64	0.34	99.3	Red Sea water column Station 192	50	27.897 N 34.507 E	PRJNA289734	SAMN04534663	LURI00000000	2651870204
Synechococcus sp. REDSEA-S01_B1	1.80	78	2216	62.76	p__Cyanobacteria (UID2143)	95.92	0.82	95.1	Red Sea water column Station 12	10	17.662 N 40.905 E	PRJNA289734	SAMN04534664	LURJ00000000	2651870193
Synechococcus sp. REDSEA-S02_B4	1.78	81	1724	62.76	p__Cyanobacteria (UID2143)	95.02	0.27	94.75	Red Sea water column Station 12	25	17.662 N 40.905 E	PRJNA289734	SAMN04534665	LURK00000000	2651870194
Unclassified gammaproteobacterium REDSEA-S03_B5	1.17	129	1411	38.75	p__Proteobacteria (UID3880)	67.34	1.22	66.12	Red Sea water column Station 12	47	17.662 N 40.905 E	PRJNA289734	SAMN04534682	LUSB00000000	2651870282
Unclassified gammaproteobacterium REDSEA-S08_B8	1.22	235	2798	51.01	p__Proteobacteria (UID3882)	55.59	0.61	54.98	Red Sea water column Station 22	200	17.996 N 39.799 E	PRJNA289734	SAMN04534683	LUSC00000000	2651870283
Unclassified gammaproteobacterium REDSEA-S09_B13	2.28	371	4019	51.81	p__Proteobacteria (UID3880)	73.03	1.22	71.81	Red Sea water column Station 22	500	17.996 N 39.799 E	PRJNA289734	SAMN04534684	LUSD00000000	2651870284
Unclassified gammaproteobacterium REDSEA-S12_B4	1.30	146	3073	38.79	p__Proteobacteria (UID3880)	71.75	4.27	67.48	Red Sea water column Station 34	50	18.58 N 40.743 E	PRJNA289734	SAMN04534685	LUSE00000000	2651870285
Unclassified gammaproteobacterium REDSEA-S14_B7	2.48	348	1860	52.09	p__Proteobacteria (UID3880)	83.9	1.37	82.53	Red Sea water column Station 34	200	18.58 N 40.743 E	PRJNA289734	SAMN04534686	LUSF00000000	2651870286
Unclassified gammaproteobacterium REDSEA-S15_B12	2.90	309	1677	52.01	p__Proteobacteria (UID3880)	89.74	1.93	87.81	Red Sea water column Station 34	258	18.58 N 40.743 E	PRJNA289734	SAMN04534687	LUSG00000000	2651870287
Unclassified gammaproteobacterium REDSEA-S21_B8	2.72	374	3048	52.02	p__Proteobacteria (UID3880)	89.65	2.44	87.21	Red Sea water column Station 91	500	20.525 N 38.781 E	PRJNA289734	SAMN04534688	LUSH00000000	2651870288
Unclassified gammaproteobacterium REDSEA-S26_B10	1.41	345	1799	52.17	p__Proteobacteria (UID3882)	54.2	1.88	52.32	Red Sea water column Station 108	200	22.046 N 37.929 E	PRJNA289734	SAMN04534689	LUSI00000000	2651870289
Unclassified gammaproteobacterium REDSEA-S27_B14	1.43	327	1742	52.05	p__Proteobacteria (UID3880)	57.13	2.44	54.69	Red Sea water column Station 108	500	22.046 N 37.929 E	PRJNA289734	SAMN04534690	LUSJ00000000	2654587903
Unclassified gammaproteobacterium REDSEA-S33_B15	1.53	372	1853	52.31	p__Proteobacteria (UID3882)	56	1.02	54.98	Red Sea water column Station 149	500	23.604 N 37.054 E	PRJNA289734	SAMN04534691	LUSK00000000	2651870290
Unclassified gammaproteobacterium REDSEA-S45_B9	2.31	340	2626	51.86	p__Proteobacteria (UID3880)	70.43	2.03	68.4	Red Sea water column Station 192	500	27.897 N 34.507 E	PRJNA289734	SAMN04534692	LUSL00000000	2651870291
