# Supplementary material for: A catalogue of 136 microbial draft genomes from Red Sea metagenomes
Source: Sci Data. 2016 Jul 5;3:160050. doi: 10.1038/sdata.2016.50 (PMC4932879; doi:10.1038/sdata.2016.50)
Supplement: Supplementary Tables [file sdata201650-s2.doc]

**Table S1**. Phylogenetically-informative bacterial marker genes.

| Marker ID | Name | Description | Length (aa) |
| --- | --- | --- | --- |
| PF02576.12 | DUF150 | Uncharacterised BCR, YhbC family COG0779 | 141 |
| PF01025.14 | GrpE | GrpE | 166 |
| PF03726.9 | PNPase | Polyribonucleotide nucleotidyltransferase, RNA binding domain | 83 |
| PF00466.15 | Ribosomal_L10 | Ribosomal protein L10 | 100 |
| PF00410.14 | Ribosomal_S8 | Ribosomal protein S8 | 129 |
| PF00380.14 | Ribosomal_S9 | Ribosomal protein S9/S16 | 121 |
| TIGR00006 | TIGR00006 | 16S rRNA (cytosine(1402)-N(4))-methyltransferase | 310 |
| TIGR00019 | prfA | peptide chain release factor 1 | 361 |
| TIGR00020 | prfB | peptide chain release factor 2 | 365 |
| TIGR00029 | S20 | ribosomal protein bS20 | 87 |
| TIGR00043 | TIGR00043 | rRNA maturation RNase YbeY | 111 |
| TIGR00054 | TIGR00054 | RIP metalloprotease RseP | 421 |
| TIGR00059 | L17 | ribosomal protein bL17 | 112 |
| TIGR00061 | L21 | ribosomal protein bL21 | 101 |
| TIGR00064 | ftsY | signal recognition particle-docking protein FtsY | 279 |
| TIGR00065 | ftsZ | cell division protein FtsZ | 353 |
| TIGR00082 | rbfA | ribosome-binding factor A | 115 |
| TIGR00083 | ribF | riboflavin biosynthesis protein RibF | 290 |
| TIGR00084 | ruvA | Holliday junction DNA helicase RuvA | 192 |
| TIGR00086 | smpB | SsrA-binding protein | 144 |
| TIGR00088 | trmD | tRNA (guanine(37)-N(1))-methyltransferase | 233 |
| TIGR00090 | rsfS_iojap_ybeB | ribosome silencing factor | 99 |
| TIGR00092 | TIGR00092 | GTP-binding protein YchF | 368 |
| TIGR00095 | TIGR00095 | 16S rRNA (guanine(966)-N(2))-methyltransferase RsmD | 194 |
| TIGR00115 | tig | trigger factor | 410 |
| TIGR00116 | tsf | translation elongation factor Ts | 293 |
| TIGR00138 | rsmG_gidB | 16S rRNA (guanine(527)-N(7))-methyltransferase RsmG | 183 |
| TIGR00158 | L9 | ribosomal protein bL9 | 148 |
| TIGR00166 | S6 | ribosomal protein bS6 | 95 |
| TIGR00168 | infC | translation initiation factor IF-3 | 165 |
| TIGR00186 | rRNA_methyl_3 | RNA methyltransferase, TrmH family, group 3 | 240 |
| TIGR00194 | uvrC | excinuclease ABC subunit C | 574 |
| TIGR00250 | RNAse_H_YqgF | putative transcription antitermination factor YqgF | 130 |
| TIGR00337 | PyrG | CTP synthase | 526 |
| TIGR00344 | alaS | alanine--tRNA ligase | 847 |
| TIGR00362 | DnaA | chromosomal replication initiator protein DnaA | 437 |
| TIGR00382 | clpX | ATP-dependent Clp protease, ATP-binding subunit ClpX | 414 |
| TIGR00392 | ileS | isoleucine--tRNA ligase | 861 |
| TIGR00396 | leuS_bact | leucine--tRNA ligase | 843 |
| TIGR00398 | metG | methionine--tRNA ligase | 530 |
| TIGR00414 | serS | serine--tRNA ligase | 418 |
| TIGR00416 | sms | DNA repair protein RadA | 454 |
| TIGR00420 | trmU | tRNA (5-methylaminomethyl-2-thiouridylate)-methyltransferase | 351 |
| TIGR00431 | TruB | tRNA pseudouridine(55) synthase | 210 |
| TIGR00435 | cysS | cysteine--tRNA ligase | 466 |
| TIGR00436 | era | GTP-binding protein Era | 270 |
| TIGR00442 | hisS | histidine--tRNA ligase | 406 |
| TIGR00445 | mraY | phospho-N-acetylmuramoyl-pentapeptide-transferase | 321 |
| TIGR00456 | argS | arginine--tRNA ligase | 569 |
| TIGR00459 | aspS_bact | aspartate--tRNA ligase | 586 |
| TIGR00460 | fmt | methionyl-tRNA formyltransferase | 315 |
| TIGR00468 | pheS | phenylalanine--tRNA ligase, alpha subunit | 324 |
| TIGR00472 | pheT_bact | phenylalanine--tRNA ligase, beta subunit | 798 |
| TIGR00487 | IF-2 | translation initiation factor IF-2 | 587 |
| TIGR00496 | frr | ribosome recycling factor | 176 |
| TIGR00539 | hemN_rel | putative oxygen-independent coproporphyrinogen III oxidase | 361 |
| TIGR00580 | mfd | transcription-repair coupling factor | 923 |
| TIGR00593 | pola | DNA polymerase I | 890 |
| TIGR00615 | recR | recombination protein RecR | 196 |
| TIGR00631 | uvrb | excinuclease ABC subunit B | 658 |
| TIGR00634 | recN | DNA repair protein RecN | 563 |
| TIGR00635 | ruvB | Holliday junction DNA helicase RuvB | 305 |
| TIGR00643 | recG | ATP-dependent DNA helicase RecG | 629 |
| TIGR00663 | dnan | DNA polymerase III, beta subunit | 367 |
| TIGR00717 | rpsA | ribosomal protein bS1 | 516 |
| TIGR00755 | ksgA | ribosomal RNA small subunit methyltransferase A | 256 |
| TIGR00810 | secG | preprotein translocase, SecG subunit | 73 |
| TIGR00922 | nusG | transcription termination/antitermination factor NusG | 172 |
| TIGR00928 | purB | adenylosuccinate lyase | 436 |
| TIGR00959 | ffh | signal recognition particle protein | 428 |
| TIGR00963 | secA | preprotein translocase, SecA subunit | 787 |
| TIGR00964 | secE_bact | preprotein translocase, SecE subunit | 57 |
| TIGR00967 | 3a0501s007 | preprotein translocase, SecY subunit | 414 |
| TIGR01009 | rpsC_bact | ribosomal protein uS3 | 212 |
| TIGR01011 | rpsB_bact | ribosomal protein uS2 | 225 |
| TIGR01017 | rpsD_bact | ribosomal protein uS4 | 200 |
| TIGR01021 | rpsE_bact | ribosomal protein uS5 | 156 |
| TIGR01029 | rpsG_bact | ribosomal protein uS7 | 154 |
| TIGR01032 | rplT_bact | ribosomal protein bL20 | 114 |
| TIGR01039 | atpD | ATP synthase F1, beta subunit | 462 |
| TIGR01044 | rplV_bact | ribosomal protein uL22 | 103 |
| TIGR01059 | gyrB | DNA gyrase, B subunit | 639 |
| TIGR01063 | gyrA | DNA gyrase, A subunit | 800 |
| TIGR01066 | rplM_bact | ribosomal protein uL13 | 141 |
| TIGR01071 | rplO_bact | ribosomal protein uL15 | 144 |
| TIGR01079 | rplX_bact | ribosomal protein uL24 | 104 |
| TIGR01082 | murC | UDP-N-acetylmuramate--L-alanine ligase | 449 |
| TIGR01087 | murD | UDP-N-acetylmuramoylalanine--D-glutamate ligase | 441 |
| TIGR01128 | holA | DNA polymerase III, delta subunit | 314 |
| TIGR01146 | ATPsyn_F1gamma | ATP synthase F1, gamma subunit | 286 |
| TIGR01164 | rplP_bact | ribosomal protein uL16 | 126 |
| TIGR01169 | rplA_bact | ribosomal protein uL1 | 227 |
| TIGR01171 | rplB_bact | ribosomal protein uL2 | 275 |
| TIGR01302 | IMP_dehydrog | inosine-5'-monophosphate dehydrogenase | 450 |
| TIGR01391 | dnaG | DNA primase | 414 |
| TIGR01393 | lepA | elongation factor 4 | 595 |
| TIGR01394 | TypA_BipA | GTP-binding protein TypA/BipA | 594 |
| TIGR01510 | coaD_prev_kdtB | pantetheine-phosphate adenylyltransferase | 155 |
| TIGR01632 | L11_bact | ribosomal protein uL11 | 140 |
| TIGR01951 | nusB | transcription antitermination factor NusB | 131 |
| TIGR01953 | NusA | transcription termination factor NusA | 340 |
| TIGR02012 | tigrfam_recA | protein RecA | 321 |
| TIGR02013 | rpoB | DNA-directed RNA polymerase, beta subunit | 1238 |
| TIGR02027 | rpoA | DNA-directed RNA polymerase, alpha subunit | 298 |
| TIGR02075 | pyrH_bact | UMP kinase | 233 |
| TIGR02191 | RNaseIII | ribonuclease III | 219 |
| TIGR02273 | 16S_RimM | 16S rRNA processing protein RimM | 166 |
| TIGR02350 | prok_dnaK | chaperone protein DnaK | 596 |
| TIGR02386 | rpoC_TIGR | DNA-directed RNA polymerase, beta' subunit | 1147 |
| TIGR02397 | dnaX_nterm | DNA polymerase III, subunit gamma and tau | 355 |
| TIGR02432 | lysidine_TilS_N | tRNA(Ile)-lysidine synthetase | 189 |
| TIGR02729 | Obg_CgtA | Obg family GTPase CgtA | 329 |
| TIGR03263 | guanyl_kin | guanylate kinase | 180 |
| TIGR03594 | GTPase_EngA | ribosome-associated GTPase EngA | 432 |
| TIGR03625 | L3_bact | 50S ribosomal protein uL3 | 202 |
| TIGR03632 | uS11_bact | ribosomal protein uS11 | 117 |
| TIGR03654 | L6_bact | ribosomal protein uL6 | 175 |
| TIGR03723 | T6A_TsaD_YgjD | tRNA threonylcarbamoyl adenosine modification protein TsaD | 314 |
| TIGR03725 | T6A_YeaZ | tRNA threonylcarbamoyl adenosine modification protein YeaZ | 212 |
| TIGR03953 | rplD_bact | 50S ribosomal protein uL4 | 188 |

**Table S2**. Phylogenetically-informative archaeal marker genes.

| Marker ID | Name | Description | Length (aa) |
| --- | --- | --- | --- |
| PF01990.12 | ATP-synt_F | ATP synthase (F/14-kDa) subunit | 95 |
| PF01866.12 | Diphthamide_syn | Putative diphthamide synthesis protein | 307 |
| PF04104.9 | DNA_primase_lrg | Eukaryotic and archaeal DNA primase, large subunit | 260 |
| PF01984.15 | dsDNA_bind | Double-stranded DNA-binding domain | 107 |
| PF02006.11 | DUF137 | Protein of unknown function DUF137 | 178 |
| PF04019.7 | DUF359 | Protein of unknown function (DUF359) | 121 |
| PF01864.12 | DUF46 | Putative integral membrane protein DUF46 | 175 |
| PF04919.7 | DUF655 | Protein of unknown function (DUF655) | 181 |
| PF07541.7 | EIF_2_alpha | Eukaryotic translation initiation factor 2 alpha subunit | 114 |
| PF13685.1 | Fe-ADH_2 | Iron-containing alcohol dehydrogenase | 250 |
| PF01269.12 | Fibrillarin | Fibrillarin | 229 |
| PF00368.13 | HMG-CoA_red | Hydroxymethylglutaryl-coenzyme A reductase | 373 |
| PF01798.13 | Nop | Putative snoRNA binding domain | 150 |
| PF00687.16 | Ribosomal_L1 | Ribosomal protein L1p/L10e family | 220 |
| PF00466.15 | Ribosomal_L10 | Ribosomal protein L10 | 100 |
| PF00827.12 | Ribosomal_L15e | Ribosomal L15 | 192 |
| PF01280.15 | Ribosomal_L19e | Ribosomal protein L19e | 148 |
| PF01157.13 | Ribosomal_L21e | Ribosomal protein L21e | 99 |
| PF01198.14 | Ribosomal_L31e | Ribosomal protein L31e | 83 |
| PF01655.13 | Ribosomal_L32e | Ribosomal protein L32 | 110 |
| PF01090.14 | Ribosomal_S19e | Ribosomal protein S19e | 140 |
| PF01282.14 | Ribosomal_S24e | Ribosomal protein S24e | 84 |
| PF01200.13 | Ribosomal_S28e | Ribosomal protein S28e | 69 |
| PF01015.13 | Ribosomal_S3Ae | Ribosomal S3Ae family | 195 |
| PF00900.15 | Ribosomal_S4e | Ribosomal family S4e | 77 |
| PF01092.14 | Ribosomal_S6e | Ribosomal protein S6e | 127 |
| PF00410.14 | Ribosomal_S8 | Ribosomal protein S8 | 129 |
| PF01000.21 | RNA_pol_A_bac | RNA polymerase Rpb3/RpoA insert domain | 112 |
| PF13656.1 | RNA_pol_L_2 | RNA polymerase Rpb3/Rpb11 dimerisation domain | 77 |
| PF01194.12 | RNA_pol_N | RNA polymerases N / 8 kDa subunit | 60 |
| PF03874.11 | RNA_pol_Rpb4 | RNA polymerase Rpb4 | 117 |
| PF01191.14 | RNA_pol_Rpb5_C | RNA polymerase Rpb5, C-terminal domain | 74 |
| PF02978.14 | SRP_SPB | Signal peptide binding domain | 104 |
| PF01868.11 | UPF0086 | Domain of unknown function UPF0086 | 89 |
| PF01496.14 | V_ATPase_I | V-type ATPase 116kDa subunit family | 759 |
| TIGR00021 | rpiA | ribose 5-phosphate isomerase A | 218 |
| TIGR00037 | eIF_5A | translation elongation factor IF5A | 130 |
| TIGR00042 | TIGR00042 | non-canonical purine NTP pyrophosphatase, RdgB/HAM1 family | 184 |
| TIGR00064 | ftsY | signal recognition particle-docking protein FtsY | 279 |
| TIGR00111 | pelota | mRNA surveillance protein pelota | 351 |
| TIGR00134 | gatE_arch | glutamyl-tRNA(Gln) amidotransferase, subunit E | 622 |
| TIGR00240 | ATCase_reg | aspartate carbamoyltransferase, regulatory subunit | 150 |
| TIGR00264 | TIGR00264 | alpha-NAC homolog | 116 |
| TIGR00270 | TIGR00270 | TIGR00270 family protein | 154 |
| TIGR00279 | uL16_euk_arch | ribosomal protein uL16 | 172 |
| TIGR00283 | arch_pth2 | peptidyl-tRNA hydrolase | 115 |
| TIGR00291 | RNA_SBDS | rRNA metabolism protein, SBDS family | 231 |
| TIGR00293 | TIGR00293 | prefoldin, alpha subunit | 129 |
| TIGR00307 | eS8 | ribosomal protein eS8 | 127 |
| TIGR00308 | TRM1 | N2,N2-dimethylguanosine tRNA methyltransferase | 375 |
| TIGR00323 | eIF-6 | putative translation initiation factor eIF-6 | 215 |
| TIGR00324 | endA | tRNA-intron lyase | 177 |
| TIGR00335 | primase_sml | putative DNA primase, eukaryotic-type, small subunit | 324 |
| TIGR00336 | pyrE | orotate phosphoribosyltransferase | 173 |
| TIGR00337 | PyrG | CTP synthase | 526 |
| TIGR00373 | TIGR00373 | transcription factor E | 162 |
| TIGR00389 | glyS_dimeric | glycine--tRNA ligase | 565 |
| TIGR00392 | ileS | isoleucine--tRNA ligase | 861 |
| TIGR00398 | metG | methionine--tRNA ligase | 530 |
| TIGR00405 | KOW_elon_Spt5 | transcription elongation factor Spt5 | 145 |
| TIGR00408 | proS_fam_I | proline--tRNA ligase | 475 |
| TIGR00422 | valS | valine--tRNA ligase | 863 |
| TIGR00425 | CBF5 | putative rRNA pseudouridine synthase | 322 |
| TIGR00432 | arcsn_tRNA_tgt | tRNA-guanine(15) transglycosylase | 637 |
| TIGR00442 | hisS | histidine--tRNA ligase | 406 |
| TIGR00448 | rpoE | DNA-directed RNA polymerase | 179 |
| TIGR00456 | argS | arginine--tRNA ligase | 569 |
| TIGR00458 | aspS_nondisc | aspartate--tRNA(Asn) ligase | 428 |
| TIGR00463 | gltX_arch | glutamate--tRNA ligase | 560 |
| TIGR00468 | pheS | phenylalanine--tRNA ligase, alpha subunit | 324 |
| TIGR00471 | pheT_arch | phenylalanine--tRNA ligase, beta subunit | 551 |
| TIGR00490 | aEF-2 | translation elongation factor aEF-2 | 720 |
| TIGR00491 | aIF-2 | translation initiation factor aIF-2 | 594 |
| TIGR00501 | met_pdase_II | methionine aminopeptidase, type II | 295 |
| TIGR00521 | coaBC_dfp | phosphopantothenoylcysteine decarboxylase / phosphopantothenate--cysteine ligase | 392 |
| TIGR00522 | dph5 | diphthine synthase | 258 |
| TIGR00549 | mevalon_kin | mevalonate kinase | 276 |
| TIGR00658 | orni_carb_tr | ornithine carbamoyltransferase | 304 |
| TIGR00670 | asp_carb_tr | aspartate carbamoyltransferase | 304 |
| TIGR00729 | TIGR00729 | ribonuclease HII | 207 |
| TIGR00936 | ahcY | adenosylhomocysteinase | 416 |
| TIGR00982 | uS12_E_A | ribosomal protein uS12 | 139 |
| TIGR01008 | uS3_euk_arch | ribosomal protein uS3 | 195 |
| TIGR01012 | uS2_euk_arch | ribosomal protein uS2 | 196 |
| TIGR01018 | uS4_arch | ribosomal protein uS4 | 162 |
| TIGR01020 | uS5_euk_arch | ribosomal protein uS5 | 212 |
| TIGR01025 | uS19_arch | ribosomal protein uS19 | 135 |
| TIGR01028 | uS7_euk_arch | ribosomal protein uS7 | 186 |
| TIGR01038 | uL22_arch_euk | ribosomal protein uL22 | 148 |
| TIGR01046 | uS10_euk_arch | ribosomal protein uS10 | 99 |
| TIGR01052 | top6b | DNA topoisomerase VI, B subunit | 488 |
| TIGR01060 | eno | phosphopyruvate hydratase | 425 |
| TIGR01077 | L13_A_E | ribosomal protein uL13 | 141 |
| TIGR01080 | rplX_A_E | ribosomal protein uL24 | 116 |
| TIGR01213 | pseudo_Pus10arc | tRNA pseudouridine(54/55) synthase | 387 |
| TIGR01309 | uL30_arch | ribosomal protein uL30 | 151 |
| TIGR01952 | nusA_arch | NusA family KH domain protein, archaeal | 141 |
| TIGR02076 | pyrH_arch | putative uridylate kinase | 222 |
| TIGR02153 | gatD_arch | glutamyl-tRNA(Gln) amidotransferase, subunit D | 405 |
| TIGR02236 | recomb_radA | DNA repair and recombination protein RadA | 311 |
| TIGR02258 | 2_5_ligase | 2'-5' RNA ligase | 180 |
| TIGR02338 | gimC_beta | prefoldin, beta subunit | 110 |
| TIGR02389 | RNA_pol_rpoA2 | DNA-directed RNA polymerase, subunit A'' | 367 |
| TIGR02390 | RNA_pol_rpoA1 | DNA-directed RNA polymerase subunit A' | 867 |
| TIGR02651 | RNase_Z | ribonuclease Z | 302 |
| TIGR03626 | L3_arch | ribosomal protein uL3 | 331 |
| TIGR03627 | uS9_arch | ribosomal protein uS9 | 130 |
| TIGR03628 | arch_S11P | ribosomal protein uS11 | 117 |
| TIGR03629 | uS13_arch | ribosomal protein uS13 | 144 |
| TIGR03636 | uL23_arch | ribosomal protein uL23 | 77 |
| TIGR03653 | uL6_arch | ribosomal protein uL6 | 170 |
| TIGR03665 | arCOG04150 | arCOG04150 universal archaeal KH domain protein | 173 |
| TIGR03670 | rpoB_arch | DNA-directed RNA polymerase subunit B | 599 |
| TIGR03671 | cca_archaeal | CCA-adding enzyme | 410 |
| TIGR03672 | rpl4p_arch | 50S ribosomal protein uL4 | 251 |
| TIGR03673 | uL14_arch | 50S ribosomal protein uL14 | 131 |
| TIGR03674 | fen_arch | flap structure-specific endonuclease | 338 |
| TIGR03677 | eL8_ribo | ribosomal protein eL8 | 117 |
| TIGR03680 | eif2g_arch | translation initiation factor 2, gamma subunit | 407 |
| TIGR03683 | A-tRNA_syn_arch | alanine--tRNA ligase | 902 |
| TIGR03684 | arCOG00985 | arCOG04150 universal archaeal PUA-domain protein | 154 |
| TIGR03722 | arch_KAE1 | universal archaeal protein Kae1 | 323 |
